# Text analysis framework for identifying mutations among non-small cell lung cancer patients from laboratory data

**DOI:** 10.1186/s12874-024-02192-8

**Published:** 2024-03-11

**Authors:** Amman Yusuf, Devon J. Boyne, Dylan E. O’Sullivan, Darren R. Brenner, Winson Y. Cheung, Imran Mirza, Tamer N. Jarada

**Affiliations:** 1 Department of Oncology, University of Calgary, Calgary, AB T2N 4N2 Canada; 2 Department of Community Health Sciences, University of Calgary, Calgary, AB T2N 4Z6 Canada; 3 Alberta Precision Laboratories, Calgary, AB T2L 2K8 Canada

**Keywords:** Text analysis, Information extraction, Syntax analysis, Lexical analysis, Context free grammar, Laboratory data, EGFR, NSCLC

## Abstract

**Background:**

Laboratory data can provide great value to support research aimed at reducing the incidence, prolonging survival and enhancing outcomes of cancer. Data is characterized by the information it carries and the format it holds. Data captured in Alberta’s biomarker laboratory repository is free text, cluttered and rouge. Such data format limits its utility and prohibits broader adoption and research development. Text analysis for information extraction of unstructured data can change this and lead to more complete analyses. Previous work on extracting relevant information from free text, unstructured data employed Natural Language Processing (NLP), Machine Learning (ML), rule-based Information Extraction (IE) methods, or a hybrid combination between them.

**Methods:**

In our study, text analysis was performed on Alberta Precision Laboratories data which consisted of 95,854 entries from the Southern Alberta Dataset (SAD) and 6944 entries from the Northern Alberta Dataset (NAD). The data covers all of Alberta and is completely population-based. Our proposed framework is built around rule-based IE methods. It incorporates topics such as Syntax and Lexical analyses to achieve deterministic extraction of data from biomarker laboratory data (i.e., Epidermal Growth Factor Receptor (EGFR) test results). Lexical analysis compromises of data cleaning and pre-processing, Rich Text Format text conversion into readable plain text format, and normalization and tokenization of text. The framework then passes the text into the Syntax analysis stage which includes the rule-based method of extracting relevant data. Rule-based patterns of the test result are identified, and a Context Free Grammar then generates the rules of information extraction. Finally, the results are linked with the Alberta Cancer Registry to support real-world cancer research studies.

**Results:**

Of the original 5512 entries in the SAD dataset and 5017 entries in the NAD dataset which were filtered for EGFR, the framework yielded 5129 and 3388 extracted EGFR test results from the SAD and NAD datasets, respectively. An accuracy of 97.5% was achieved on a random sample of 362 tests.

**Conclusions:**

We presented a text analysis framework to extract specific information from unstructured clinical data. Our proposed framework has shown that it can successfully extract relevant information from EGFR test results.

**Supplementary Information:**

The online version contains supplementary material available at 10.1186/s12874-024-02192-8.

## Introduction

Real world studies leveraging data from electronic health records and administrative data can have a considerable impact on clinical practice [1, 2]. However, these studies are often limited by a lack of laboratory data, which are becoming increasingly important with the rise in targeted therapies and biomarkers of treatment response. One of the reasons for the lack of this data in real-world studies is that the data is typically stored in an unstructured manner as free text or notes. Information Extraction (IE) of unstructured clinical and laboratory data can augment real-world datasets and can lead to more complete analyses. One of the most prevalent methods for IE is Natural Language Processing (NLP), as initially explored in the context of medical study by Spyns in 1996 [3]. Since these early studies, several applications have evaluated NLP using various rule-based approaches to extract clinical patient data from mammography reports and hospital records of diabetic patients [4].

In this study, we applied a formalized method of extracting Epidermal Growth Factor Receptor (EGFR) test results from free text, structured data. Among Non-Small Cell Lung Cancer (NSCLC) patients a notable proportion of patients will have a mutation in the EGFR gene. Molecular therapies known as tyrosine kinase inhibitors (TKI) have been developed to target these mutations within the cancer cells. These treatments have been shown to have a meaningful impact on cancer outcomes in these patients. In many health data settings, the results from the tests come from non-standardized reports and free-text results from disperse laboratory reports. We aimed to harmonize these EGFR data and develop a systematic approach to extract a large volume of these free-text testing data. The original data were provided by the Alberta Precision Laboratories. The main goal was to extract the EGFR test result which is the identification or detection of a gene mutation from advanced NSCLC.

A rule-based method for IE can start with Lexical Analysis. Lexical Analysis in computer science is the first process in a compiler which takes the input text file, groups characters into lexemes and converts the lexemes into a sequence of tokens [5]. Lexemes are a sequence of characters that match the pattern of a token while a token is a symbol representing a lexical unit. For example, the string of characters “*integer*” is a lexeme that matches the integer token represented by the symbol“*INT*”. The role of the Lexical Analysis stage also includes cleaning the text input and removing white space for the next stage. That text is then normalized and key words are grouped into lexemes and converted into tokens. Key words are an important set of words in the input data which will dictate the data representation of the original input. These key words in the context of our study can include mutation locations, test results, and conjunctions. The tokenized input data is then passed into the next stage which is the Syntax Analysis stage.

The Syntax Analysis stage determines the syntax structure of the text based on a grammar. This stage is also referred to as the parsing stage, where the tokenized input is parsed based on our generated grammar. Context Free Grammars were first introduced by Noam Chomsky [6] as a formal way to describe the composition of sentences and words in natural language. For example, the English language constitutes of verbs, nouns, adjectives, and prepositions and grammar would determine the relationship and structure between them. In the field of computer science, Context Free Grammars are used to describe the structure of programming languages [7] for parsing and compilation. Context Free Grammars can be described as a set of production rules, variables, and terminals. Production rules consist of a variable, an arrow, and a combination of variables and terminals on the left-hand side. They are often called substitution rules as they dictate what the variables are replaced with. Variables are considered a set of symbols which can be replaced by the set of rules. Terminals are non-variable characters which cannot be replaced. Formally, a Context Free Grammar can be described as follows [7]:

A context free grammar G is a 4-tuple *G* = (*V,Σ,R,S*) in which:

1. *V is the finite set of variables where they represent the phrases of the language*.
2. Σ is a finite set of terminals disjoint from *V*. They make up the actual characters of the language.
3. *R* is the finite set of production/substitution rules. It a relation of the form *V* × (V ∪ Σ)*.
4. *S* is the start symbol/variable where *S* ∈ *V*

Herein, we review approaches and applications of NLP to augment the laboratory data via IE. Following our review, we include a detailed description of our methods, results, limitations, and possible future applications.

## Related work

In the context of IE, the information to be extracted is mostly made of free text data [8, 9]. The methods employed to perform IE consist of rule-based methods and NLP/Machine Learning (ML) based methods. Rule-based methods use patterns to extract the information like the Context Free Grammar mentioned previously or regular expressions. We will discuss these processes and how they tie into our study.

Mykowiecka et al. [4] discuss the IE of Polish mammography reports and hospital records of diabetic patients. Even though the field of biomedical data extraction contains many ML and statistical methods to extract data, this group used a rule-based system for the initial IE due to the complex nature of the medical records and the observation that there is no adequate annotated medical corpora in Polish for ML methods. As for their rule-based method, the first step was the pre-processing step needed for raw data which includes spell-checking and converting to a proper format for analysis. A morphological analyzer was then used to bring the words to their roots for the following grammar. The grammar extracted the data and then the templates were inserted into a relational database. Due to the complexity of most Electronic Health Records (EHRs), the application of the grammar was not done until the post-processing phase. The evaluation was done based on precision and recall of a sample of EHRs, of which above 80% was achieved for both measures. Precision and recall were defined as follows:

$$\text{Precision}=\left(\frac{phrases\;correctly\;recognized}{all\;phrases\;recognized}\right)$$

$$\text{Recall}=\left(\frac{phrases\;correctly\;recognized\;representing\;the\;feature}{all\;phrases\;representing\;the\;feature}\right)$$

Shivade et al. [10] conducted a review of the different approaches used to identify and extract patient phenotype cohorts using EHRs and these included rule-based systems, NLP systems, statistical analyses, data mining, machine learning, as well as hybrid systems. The review consisted of papers published from 2010–2012 in 4 specific journals: Journal of American Medical Informatics Association*,* Journal of Biomedical Informatics*,* Proceedings of the Annual American Medical Informatics Association Symposium*, and* Proceedings of Clinical Research Informatics Conference*.* Of the 97 papers, 46 used NLP, 41 used statistical analysis /data mining/machine learning, 24 used rule-based and 22 were hybrid. The analysis of the rule-based systems mentioned that some studies modified their rules manually after analysis to account for errors while others attempted to automatically generating these set of rules. The paper then concluded that “rule-based systems are easy to interpret, fast to implement, and give good results on limited datasets”.

Meystre and Haug [11] proposed an NLP approach to extract potential problem list entries from free-text EHRs. The problem list was defined as a collection of all patient medical problems. The authors combined the UMLS MetaMap Transfer (MMTx) [12] and a negation detection algorithm called NegEx [13] as the NLP system. The pre-processing step of their methodology goes over section detection, sentence detection, and disambiguation.

Liu et al. [14] proposed an IE framework for cohort identification that is knowledge-driven and developed under Unstructured Information Management Architecture (UIMA). The paper discusses section detection as well as contextual information which are properties that pertain to a certain condition such as negation, temporality, and experiencer. In this paper, UIMA was used to implement systems for processing unstructured content. The UIMA pipeline starts with accessing the documents and converting them to the UIMA object, a Common Analysis Structure (CAS). The CAS object is then brought through the processing pipeline by adding important annotations and final processing for later analyses.

## Methods

### Software used

The software used for this study was version 3.6.3 of the R language [15]. All methods were performed in accordance with the relevant guidelines and regulations or declaration of Helsinki.

### Data preparation

**Fig. 1:**
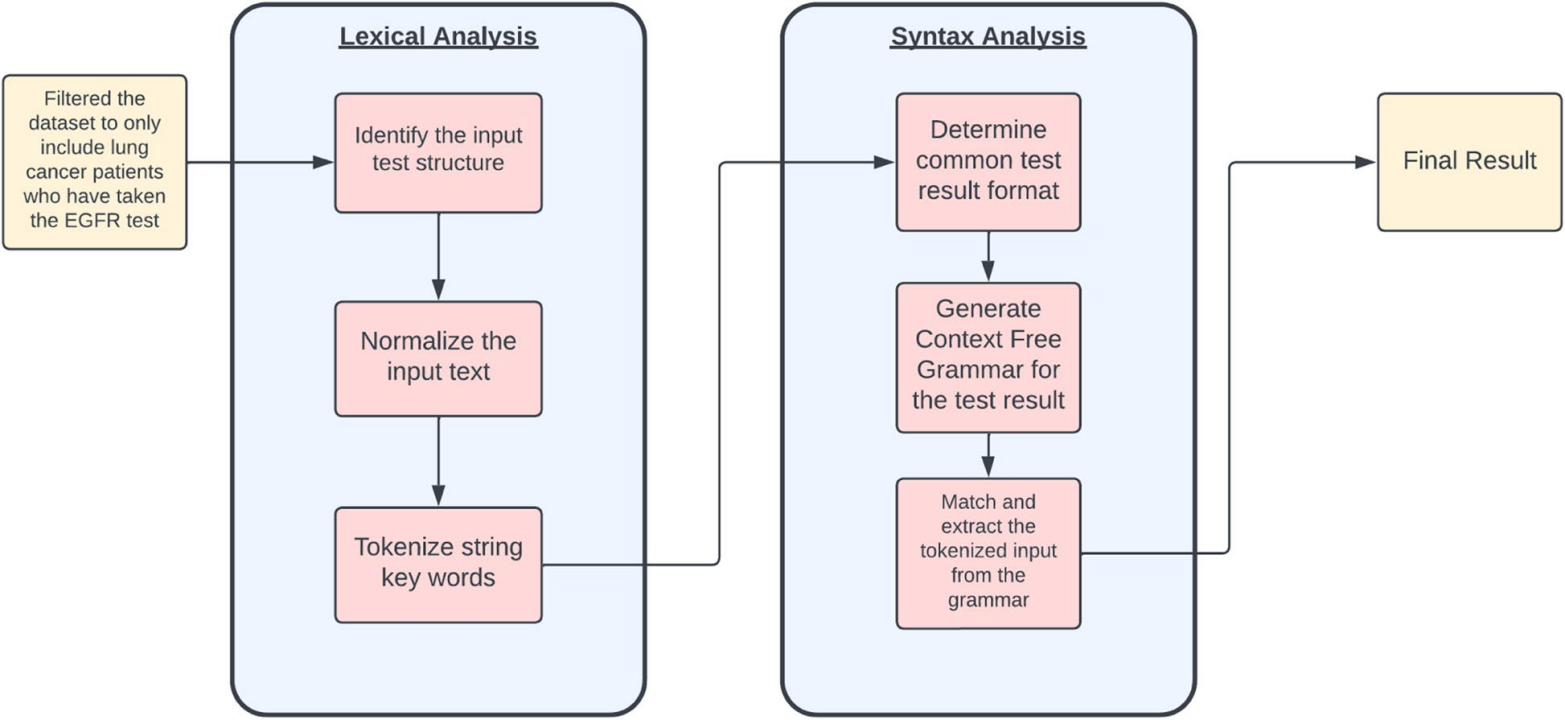
The process for Syntax and Lexical analysis to achieve deterministic extraction of data from Epidermal Growth Factor Receptor (EGFR) test results

The data used in this study were provided by the Alberta Precision Laboratories and consisted of two main datasets: Northern Alberta Dataset (NAD) and Southern Alberta Dataset (SAD). The prepared data covers all of Alberta and is completely population-based. As the data were collected through different processes within the province of Alberta, there were some differences between the two datasets. Data preparation was performed before the text analysis to filter relevant medical records. Both the NAD and SAD datasets contained patient information as well as a unique test result identification for each row. Only Alberta patients as well as patients without missing identifying information were included in the initial screening. What differed between the NAD and SAD data was how the results were stored. The SAD data stored the test results in 4 different columns: “TEST_NAME”, “TEST_TASK”, “RTF_RESULT”, and “RESULT_SHORT”. The “TEST_NAME” column contained the “EGFR Result” and the “TEST_TASK” contained “EGFR (Interpretation)” or “EGFR (Qualitative)”. The “RTF_RESULT” column included the test result in Rich Text Format, while the “RESULT_SHORT” column included an immediate result of the test, but was lacking in specificity (identified an EGFR mutation, but not the exact location). Since the focus of this study was only on patients who had EGFR testing, SAD test results that contained EGFR or EGFR Mutation Assessment/Assay in either the “TEST_NAME”, “TEST_TASK”, or “RTF_RESULT” column were extracted. Due to the lack of specificity in the “RESULT_SHORT” column we opted out of using it as a data extraction method as it would not generalize to extracting data from the NAD data. Unlike the SAD data, NAD only had one column containing the test result under the “RESULTS” column. The same criteria used in the SAD filter were used in the NAD data. Figure 1 depicts a summary of our process taken to determine EGFR mutation results using data from both the NAD and SAD datasets.

As mentioned previously, the stored free text data of the EGFR test result had a structure to it. Figure S1 in the Supplementary Material shows an example of “Not Detected” free text test result from the SAD dataset. The free text data had identifiable section headers, relevant paragraphs, and most importantly a test result section, which is demonstrated in Fig. S2 in the Supplementary Material**,** where the Rich Text Format data is viewed through a Microsoft Word document. NLP and ML methods were therefore not required to extract the main relevant data (i.e., the test result).

### Lexical analysis

**Fig. 2:**
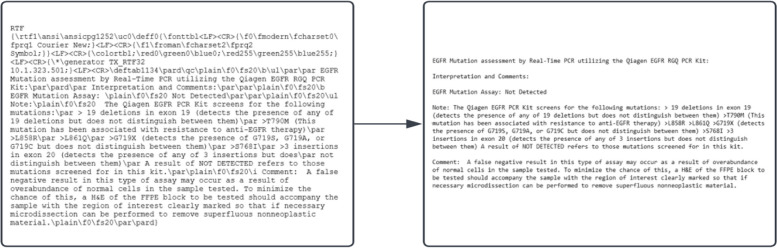
A transformation applying the striprtf function on the raw, free text EGFR data to readable text

The lexical analysis stage of the method turned the free text EGFR test result data into tokenized results. In the case of the SAD data, the Rich Text Format (RTF) input was first converted into readable plain text format by using the “striprtf” R package [16]. This made further analysis more manageable as the program did not need to account for unnecessary RTF syntax. The transition can be observed in Fig. 2.

Normalization of the input was then required to simplify the variations in the free text. The bare minimum of the normalization, such as removing trailing and leading white space and bringing text to lowercase, was applied for our purposes.

Observing the result in Fig. 2 as well as Fig. S2 in the supplementary section, it was evident that the main sections of an EGFR test for the SAD dataset could be identified. The test results had explicit section heading that need to be split in order to identify the EGFR test result. An exact matching rule was employed by our algorithm to identify the different sections in the EGFR document. The sections identified were “Interpretation and Comments:”, “EGFR Mutation Assay:”, “Note:”, and “Comment:”.

The final step of the Lexical Analysis stage was to tokenize string key words into tokens. From analyzing a sample of data, we observed that the EGFR test result remained in the “Interpretation and Comments:” and “EGFR Mutation Assay:” section of the input data. The “Note” and “Comment” section of the test did not have much differentiation between individual tests and were not analyzed further. As such the “EGFR Mutation Assay” section, which was apparent in the SAD data, was used as the main source of analysis. For the NAD data, the same process was applied in identifying the sections and the result of the test was in a section labeled “Result:”. From this step we observed the following general test result from both NAD and SAD data:

- The EGFR mutation was “Not Detected”.
- The EGFR mutation was “Identified” in one location.
- The EGFR mutation was “Identified” in two or more locations.
- There was insufficient DNA for the test.

Locations in this context correspond to one of the following: Exon 19, Exon 20, T790M, L858R, L861Q, G719X, S78I. A result of “Not Detected” in the EGFR test means that none of the hotspot mutations listed above were identified, whereas a result of “Identified” means the specific mutation was detected. Our study does not need to consider the level of contextual information as discussed by Meystre and Haug [11] as the EGFR medical data presented is not as complex. The negation aspect of an EGFR test condition varies from just “negative” to “not detected”. Some results included “negative” and “positive” instead of “Not Detected” and “Identified”. These result key words were converted in the tokenization stage. There were also specific results attached to certain mutations such as the “Deletions” in Exon 19 and the “Insertions” in Exon 20.

After identifying the language used for the general test results, a reduced dictionary was made for the purpose of tokenizing key words. This dictionary included key words from the different genomic locations screened for in the test, the result of the test, and whether there was insufficient DNA. The tokenization step was then applied on the normalized result. This step converted key words such as “Exon 19” into “exon_19_location”, “Deletion” into “deletion_specific_result”, and “Identified” into “identified_result”. Conjunctions such as “and” as well as the comma “,” were also tokenized to account for multiple mutation locations in the result. Other intermediary English words were ignored in the tokenization process.

### Syntax analysis

The second step of our method involves syntax analysis and using the tokenized result from the first step to extract a result. Since the EGFR test result was consistent and simple between all entries, a Context Free Grammar (CFG) was generated to match the result. The simplified structure of the test result makes it a good application for rule-based methods such as the CFG. This method reduces the total number of derivations one can get from the test result. The following is a simplified CFG of the rules used in the actual extraction.

The context free grammar G = (*V*,Σ,*R*,*S*) where:

1. V the finite set of variables *V* = {*S, Location, Result, Conjunction, Specific_Result*}.
2. Σ the finite set of terminals Σ = {Exon 19,Exon 20,T790M,L858R,L861Q,G719X,S768I}.
3. *R* the finite set of production\/substitution rules:
  *S* → *Location Result* | *Location Conjunction S* | *S Conjunction Location*
  *Location* → *Location Specific_Result* | *Specific_Result Location*
  *Conjunction* → *and* | ,
  *Result* → *Identified* | *Not Detected*
  *Specific*_*Result* →(.^*) | *Deletion* | *Insertion* | ε
4. *S* is the start symbol/variable.

**Fig. 3:**
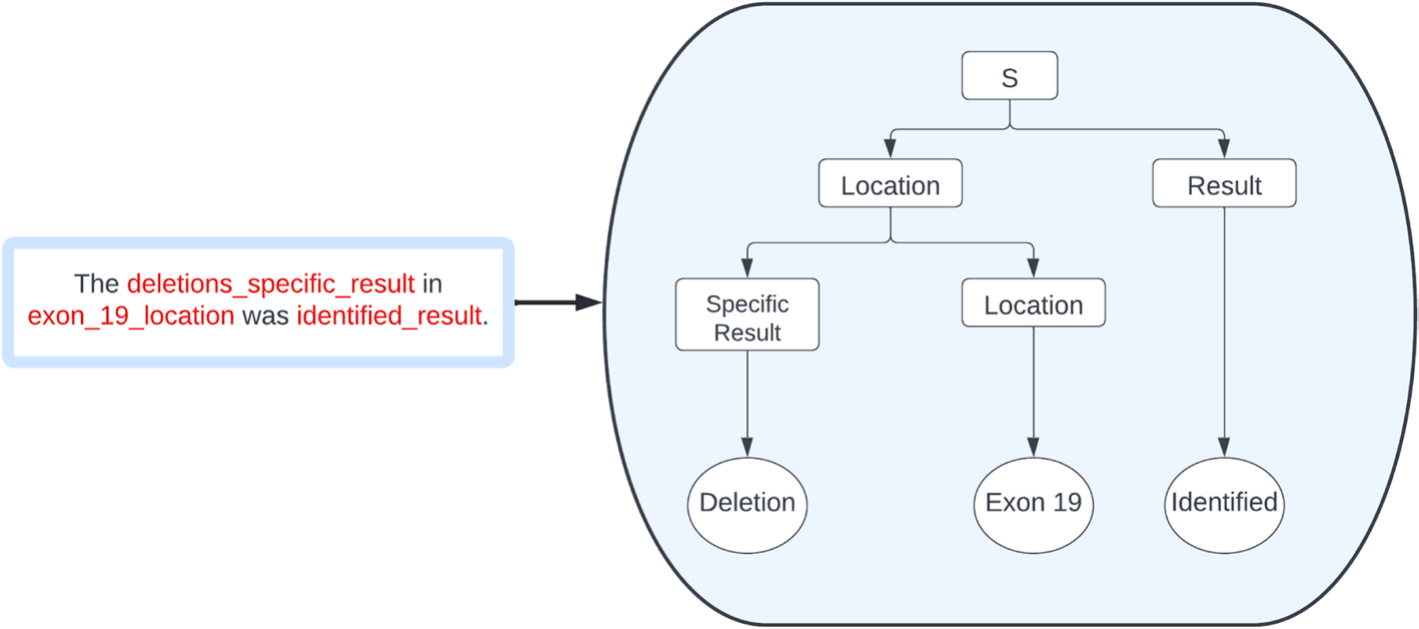
An example of applying the Context Free Grammar on a sentence from a tokenized EGFR test result to produce a parse tree

Figure 3 demonstrates an example of the proposed grammar where the original EGFR test result *“The deletions in Exon 19 was identified”* was tokenized and converted. The grammar then matched the tokenized words to generate the parse tree on the right. The terminals on the leaves of the parse tree are the result of the EGFR test. This grammar was then applied to every tokenized test result to extract the relevant data.

### Algorithm accuracy

As for evaluating the proposed algorithm accuracy, we use a sample comparison between correctly and incorrectly extracted data. A sample of EGFR tests is randomly selected, reviewed, and checked whether the relevant data is extracted or not. The random sample was identified given a set mean and standard deviation (i.e., μ = 0 and σ = 1) such that $X\sim N(\mu ,{ \sigma }^{2})$.

The following is the criteria being used to measure if the algorithm correctly extracts the data:

- If the sample detected an EGFR mutation, then the algorithm would correctly identify which location was detected.
- If the sample did not detect an EGFR mutation, then the algorithm would extract “Not Detected” from the result.

### Linkage with cancer registry

Finally, we link the results with the Alberta Cancer Registry (ACR). The ACR captures information on all individuals diagnosed with cancer within the province of Alberta, Canada.

## Results

The SAD original data set contained a total of 95,854 row entries collected over 2013 to 2019. Each row was a test result that can be identified by one unique ID called the result ID. Patients were then filtered by their Unique Lifetime Identifier (ULI) and restricted to only have lung cancer patients for this analysis, which came to a total of 16,934 row entries remained.

Initially, empty ULIs were included in the result as there was the possibility that test results could be linked to existing patients.

**Fig. 4:**
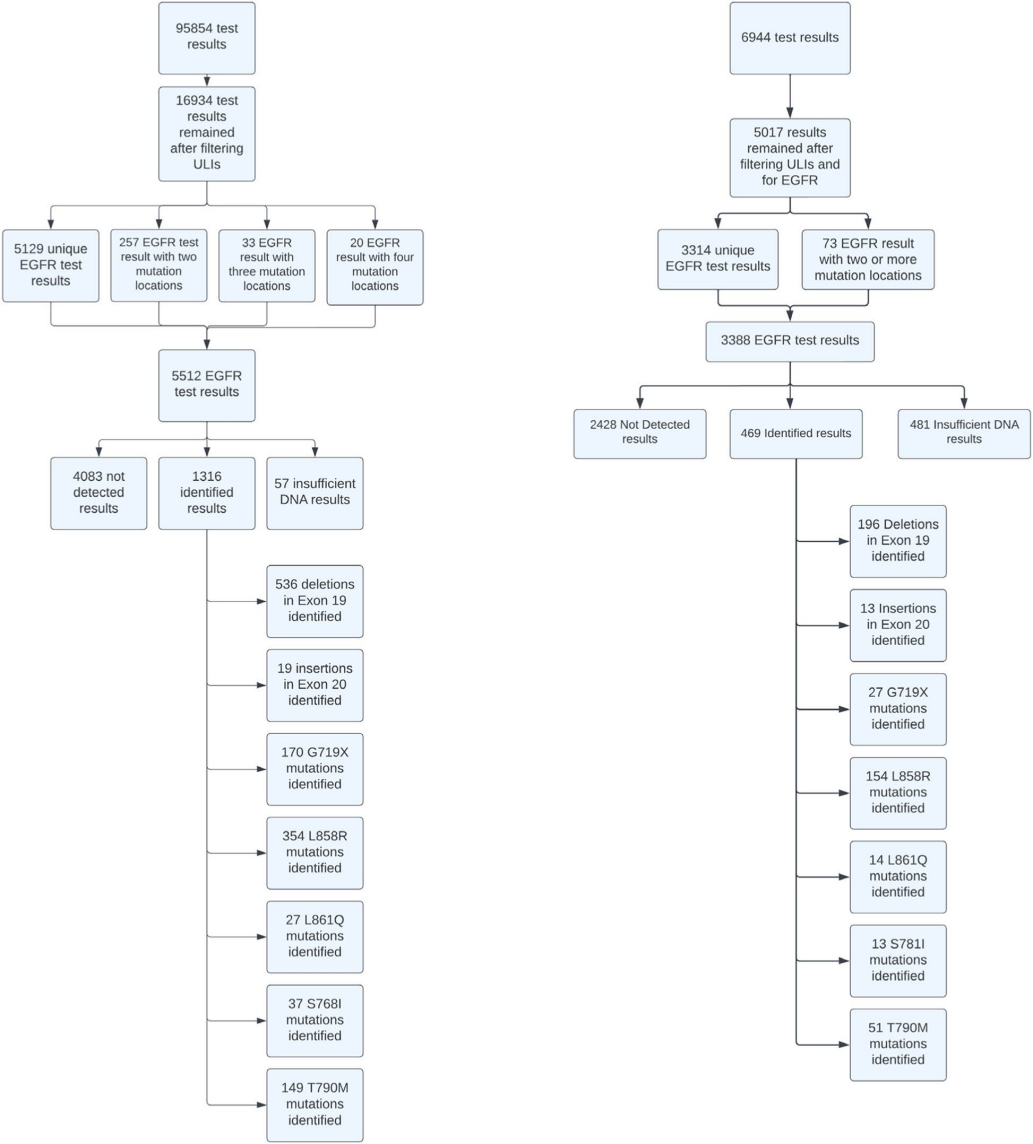
A breakdown of SAD data (left) and NAD data (right) from the original data entries to the final EGFR results

The key word “EGFR” was used to filter row entries by checking the “RTF_RESULT” and “RESULT_SHORT” columns. This resulted in 6933 rows left in the result. Upon further analysis of the “RESULT_SHORT” column, it was apparent that the intersection of row entries with “EGFR” did not yield a relevant result. As such, “EGFR” on the “SPECIMEN_ACCN”, “SPECIMEN_SOURCE”, “TEST_NAME”, “TEST_TASK”, and “RTF_RESULT” columns were then used to filter the result further as we were interested in the result of the test which came after “EGFR Mutation Assay: …”, which brought the pool of entries to 5197. We then utilized the algorithm outlined in the methods section and applied it on the data set. The “RTF_RESULT” was used as the original input and the relevant data was extracted from it. A diagram of the breakdown of the SAD results can be observed in Fig. 4. Our methodology yielded 5129 unique EGFR test results from the final 5512 row entries as some test results had more than one identified mutation location. Out of the 5512 row entries 4083 test results did not detect an EGFR mutation, 1316 identified EGFR mutations, and 57 insufficient DNA test results. As for the 1316 identified mutations 536 detected deletions in Exon 19, 354 test results had L858R mutation, 170 test results had G719S/G719A/G719C (QIAGEN® EGFR PCR Kit (QIAGEN Manchester Ltd., UK) assay detects the presence of G719S, G719A, or G719C, but does not distinguish between them), 149 with T790M, 37 with S768I, 27 with L861Q, and 19 with identified insertions in Exon 20. There was a total of 310 test results with identified mutations in more than one location with 257 in two locations, 33 in three locations, and 20 in 4 locations. Of those 310 test results with identified mutations in more than one location of the gene, there were 21 unique permutations and combinations of the locations. The majority included 80 test results with deletions in Exon 19 and mutations in T790M, 53 test results with concurrent L858R and T790M mutations, and 18 test results with G719X and S768I.

As for the NAD dataset, it originally contained 6944 row entries. Unlike the SAD patient data, the NAD row entries were all unique patients. The same filtering of lung cancer patients was conducted and resulted in 5017 unique patients. A figure of these results can be observed in Fig. 4. Out of that cohort, the same 5017 patients had mentioned the term “EGFR” in the “RESULTS” column of the dataset. After applying our methodology on the NAD data, a total of 3388 row entries were obtained with 3314 unique EGFR tests. Out of the total 3388 test results, 2428 test results did not detect an EGFR mutation, 469 did identify a mutation, and 481 test results had insufficient DNA (this result was labeled slightly different than the SAD “insufficient DNA” as these were denoted as “test cancelled due to insufficient DNA”). From the 469 identified mutations there were 196 identifications of deletions in Exon 19, 154 test results had L858R, 51 had T790M, 27 had G719X, 14 had L861Q, 13 had S768I, and 13 results had deletions in Exon 20. Of the 72 EGFR test results that included more than one mutation, location 32 of the tests had identified deletions in Exon 19 and T790M mutation, 19 tests identified T790M and L858R mutations, and less than 10 tests identified mutations in G719X and S768I.

Next, a total of 8900 EGFR tests were used in the study. Due to resource constraints, we were able to conduct comprehensive chart reviews on a total of 362 patients. We therefore took a simple random sample of 362 patients (i.e., 4.06%) from the total population of 8900 patients. The test results of the 362 patients were then manually reviewed and verified. As shown in Table 1, our proposed algorithm managed to correctly extract data from 353 out of the 362 samples with 97.5% accuracy. However, all the samples that detected an EGFR mutation were correctly extracted. This amounted to a total of 35 samples, 4 of which detected an EGFR mutation at 2 locations. Among those who underwent EGFR testing and got an EGFR mutation detected, the algorithm had an AUC of 100% with 100% sensitivity and 100% specificity. For the 9 samples which did not have their data correctly extracted, they were all cancelled tests which the algorithm extracted “Not Detected” as the result of the test. A total of 10 samples out of the 362 had an extra comment added to the sample which was not a definitive test result. There were 9 comments that mentioned low tumor cellularity for a “Not Detected” test result. One of the comments made by the clinician mentioned that the result of a “Detected” EGFR test was at the cut off at identifying a T790M mutation. Although these comments did add context and insight to the test result, they were out of the scope of the data to be extracted.

**Table 1:** Results of the model evaluation based on the random samples review

		Predicted Results			
		EGFR Detected	EGFR Not Detected	Inconclusive	Not Tested
Actual Results	EGFR Detected	35	0	0	0
	EGFR Not Detected	0	308	0	0
	Inconclusive	0	10	0	0
	Not Tested	0	9	0	0

**Fig. 5:**
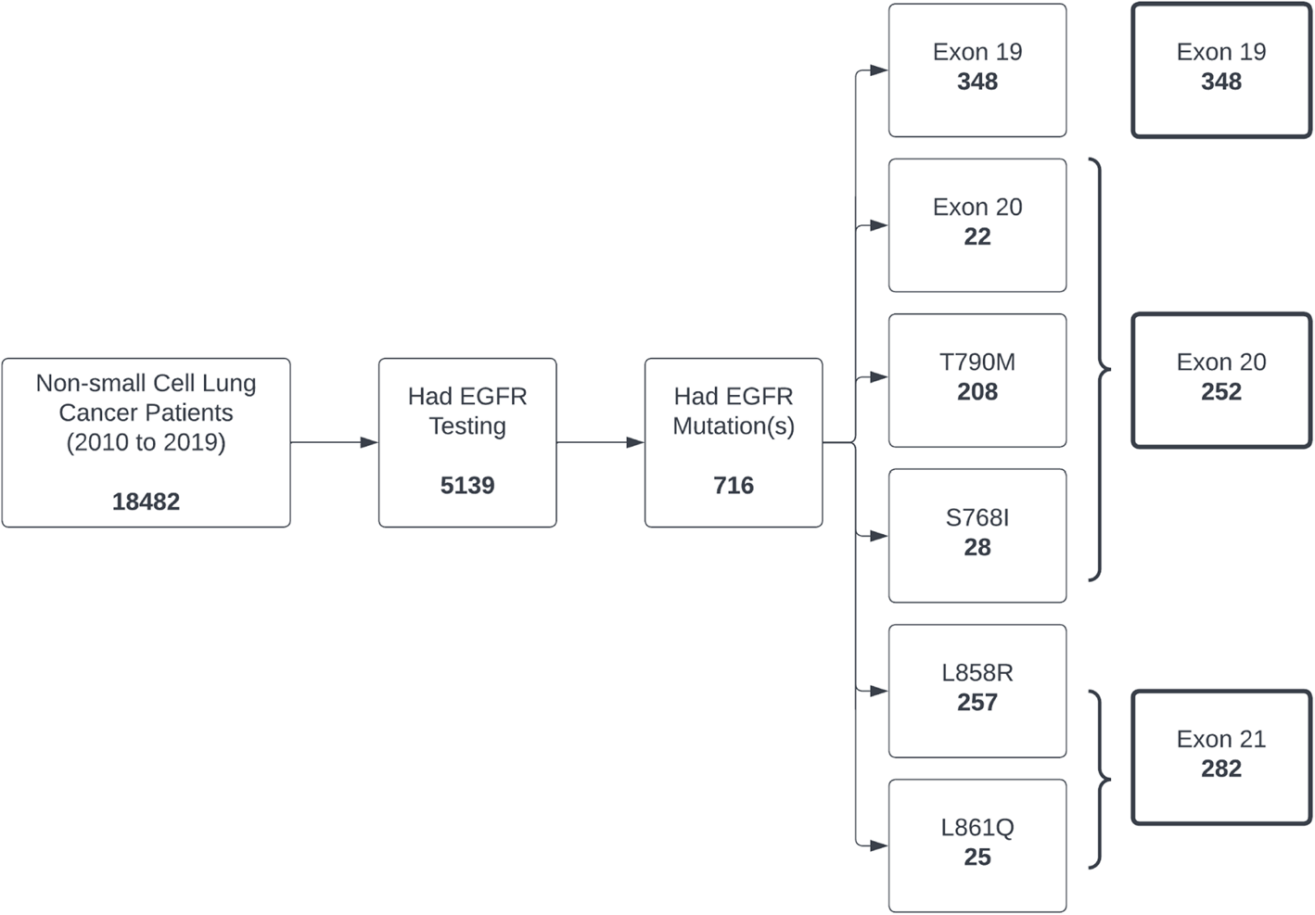
EGFR mutation results linked with the Alberta Cancer Registry data

Finally, we found out that 5139 of 18,482 non-small cell lung cancer patients in Alberta from 2010–2019 had EGFR testing. As shown in Fig. 5, 716 out of 5139 patients had EGFR mutations. A total of 348 had the Exon 19 mutation, 252 had the Exon 20 mutation (Exon 20, T790M, S768I), and 282 had the Exon 21 mutation (L858R, L861Q).

## Discussion

Overall, our extraction process proved highly effective in retrieving “Detected” test results by amalgamating data from various sources. This success can be attributed to the generally organized format in which the test results were documented, resulting in a grammar that was easily discernible and manageable. As show in Table 2, our proposed model outperformed other approaches that used machine learning analysis for electronic health records.

**Table 2:** The performance of the proposed model in comparison with other approaches that used machine learning analysis for electronic health records

Method	Accuracy
Proposed Model	97.5%
Ganesan and Subotin [17]	93.32%
Haug et al. [18]	90.90%
Apostolova et al. [19]	79%
Jancsary et al. [20]	96.48%

When comparing the accuracy of our model to the other Even among the 362 samples, where 9 test results were not initially extracted accurately, our process could rectify these discrepancies. The missed results exhibited a consistent structural pattern, which could be seamlessly integrated into the original Context-Free Grammar (CFG). However, it’s worth noting that manually augmenting the CFG could become laborious as the complexity of input data increases.

For optimal results using our proposed method, one must grasp how the test results or medical text are stored in the source data. Specifically, the SAD data required conversion from RTF to plain text. Given our focus on EGFR test results, we had a solid understanding of the formatting structure for such tests. This comprehension of the structure serves as a prerequisite for generating the necessary rules and grammar to extract the data accurately. In the event of a new test result structure that deviates from the grammar rules established in the methodology section, a manual addition of the new rule to the grammar would be imperative to capture the test result. This limitation is evidenced by the algorithm analysis, where 9 results out of 362 were identified as “Test Cancelled” due to the absence of a rule accounting for such outcomes in the original grammar.

Nevertheless, this limitation can be surmounted through the application of natural language processing or machine learning techniques, enabling the identification of patterns in the original data and the subsequent generation of appropriate grammar rules [11]. This approach affords greater flexibility in handling complex scenarios where a test result may encompass more than four potential outcomes. In our specific application, where the grammar was relatively compact and there was minimal deviation among normalized tests, the manually devised grammar rules sufficed for extracting the data.

This information extraction method can be extended to data with a similar organizational structure, necessitating a clearly discernible section housing the relevant information for extraction. The syntax and grammar structure of the target information should remain consistent across different electronic records.

In terms of future developments, an additional rule must be integrated into the method proposed in this study to account for “Test Cancelled” results and offer a more precise representation of the data. Subsequent efforts could also explore the extraction and analysis of clinician notes pertaining to the tests. This would further enhance the depth and comprehensiveness of the extracted information, contributing to a more comprehensive understanding of the underlying data.

## Conclusion

With more electronic health records becoming available, effective information extraction methods can make analyzing the data contained within these records more manageable. In this paper we presented a method to extract specific information from EGFR test results. Although effective in the context of the EGFR tests and similarly structured EHRs, future applications can and should make use of NLP and ML techniques to account for rising complexity within these records. Many additional genomic test results are stored in a similar fashion in Alberta and other North American data repositories. These data can be extremely impactful for evaluating the effect of targeted therapies and related testing on outcomes in cancer patients. Additional systematic efforts such as what we have developed are needed to increase data liberation and improve subsequent patient management and evaluation.

### Supplementary Information

**Supplementary Material 1. **

## Data Availability

Data presented in this study are aggregate-level data, individual level data are not publicly available due to Canadian data privacy laws governing personal health information.

## Abbreviations

ACR: Alberta Cancer Registry
CAS: Common Analysis Structure
CFG: Context Free Grammar
EGFR: Epidermal Growth Factor Receptor
EHRs: Electronic Health Records
IE: Information Extraction
ML: Machine Learning
MMTx: MetaMap Transfer
NAD: Northern Alberta Dataset
NLP: Natural Language Processing
NSCLC: Non-Small Cell Lung Cancer
RTF: Rich Text Format
SAD: Southern Alberta Dataset
TKI: Tyrosine Kinase Inhibitors
UIMA: Unstructured Information Management Architecture
ULI: Unique Lifetime Identifier
