# Supplementary material for: Text analysis framework for identifying mutations among non-small cell lung cancer patients from laboratory data
Source: BMC Med Res Methodol. 2024 Mar 11;24:63. doi: 10.1186/s12874-024-02192-8 (PMC10926579; doi:10.1186/s12874-024-02192-8)
Supplement: Supplementary file 1 — Supplementary Material 1. [file 12874_2024_2192_MOESM1_ESM.docx]

# Supplementary Material


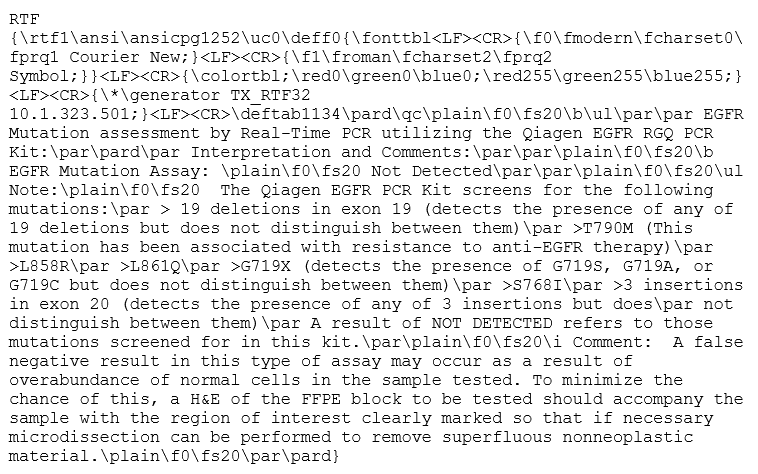


**Figure S1: An example of “Not Detected” free text test result from the SAD dataset.**


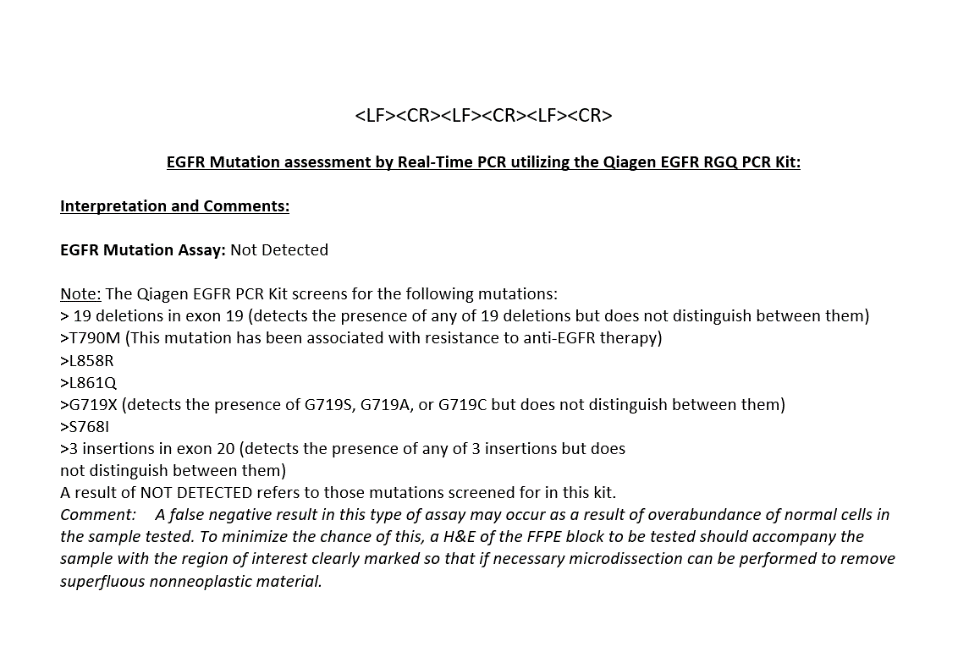


**Figure S2: Rich Text Format data from the SAD dataset viewed through a Microsoft Word document.**
